# Study on the Mechanism Between Weld Microstructure and Crack Tie Opening Displacement Fracture Toughness of the Steel Catenary Riser

**DOI:** 10.3390/ma18010176

**Published:** 2025-01-03

**Authors:** Yuxi Cao, Shubiao Yin, Ba Li, Shujun Jia, Yuan Li, Yuqin Qin, Rui Hong, Kangxin Shuai

**Affiliations:** 1 Faculty of Metallurgical and Energy Engineering, Kunming University of Science and Technology, Kunming 650031, China; 18625798528@163.com (Y.C.); yinshubiao@kust.edu.cn (S.Y.); 15121273974@163.com (Y.Q.); 15105633440@163.com (R.H.); kangxinshuai99@163.com (K.S.); 2 Engineering Steel Institute, Central Iron and Steel Research Institute, Beijing 100081, China; balicugb@sina.com (B.L.); liyuan104029@163.com (Y.L.)

**Keywords:** steel catenary riser, straight pipe weld, acicular ferrite, grain boundary polygonal ferrite, side lath ferrite, fracture toughness

## Abstract

Fracture toughness is an important index related to the service safety of marine risers, and weld is an essential component of the steel catenary risers. In this paper, microscopic structure characterization methods such as scanning electron microscopy (SEM) and electron back scatter diffraction (EBSD), as well as mechanical experiments like crack tip opening displacement (CTOD) and nanoindentation, were employed to conduct a detailed study on the influence of the microstructure characteristics of multi-wire submerged arc welded seams of steel catenary riser pipes on CTOD fracture toughness. The influence mechanisms of each microstructure characteristic on fracture toughness were clarified. The results show that the main structure in the weld of the steel catenary riser is acicular ferrite (AF), but there is also often side lath plate ferrite (FSP) and grain boundary ferrite (GBF). With the increase in the proportion of FSP and GBF in the weld microstructure, the CTOD fracture toughness of the weld decreases gradually. The weld AF is a braided cross arrangement structure, and most of the grain boundary orientation difference is higher than 45°. The effective grain size refinement of AF can effectively prevent crack propagation and significantly improve fracture toughness. GBF is distributed along proto-austenitic grain boundaries PAGB, and the large hardness difference between the GBF and the AF matrix weakens the grain boundary. Cracks can easy be initiated at the interface position of the two phases and can propagate along the GBF grain boundary, resulting in the deterioration of toughness. Although the hardness of FSP is between that of GBF and AF, it destroys the continuity of the overall weld microstructure and is also unfavorable to toughness.

## 1. Introduction

The steel catenary riser is an important part of the deep-sea oil and gas production system. It is made of welded pipe/seamless pipe connection. In harsh service environments, the steel catenary riser will face complex working conditions such as surge, vortex-induced vibration, and variable load [1,2]. In the process of steel pipe manufacturing, welding residual stress, uneven structure, and mechanical properties will inevitably occur in the weld area, which makes it easy to produce minor defects in the service process of welding [3,4,5,6]. These defects have no obvious plastic deformation on the macroscopic scale, and the deformation is mainly concentrated in the crack tip area. In the low-temperature environment, the rapid expansion of these cracks leads to a sharp reduction in the life of the pipeline [7]. Therefore, the research on the anti-crack propagation ability of weld metal has gradually attracted people’s attention. The crack tip opening displacement (CTOD) experiment can be used to determine the fracture toughness of the full thickness of the welded joint because the CTOD experiment can exclude the crack initiation energy and accurately detect the critical crack size and critical load; thus, the fracture toughness of the material containing small defects can be evaluated [8,9].

The welded joints of modern pipeline steel usually adopt the design principle of “equal strength” or “over-strength” matching. In order to ensure the good strength and toughness of the steel catenary riser, higher requirements are put forward for the fracture toughness of the weld seam [10]. Acicular ferrite (AF) is an ideal structure for the weld metal of pipeline steel because of its excellent strength and toughness. However, AF in the weld is a mesothermal phase transition product of intracrystalline nucleation, its formation mechanism is similar to that of granular bainite and bainitic ferrite, and its formation temperature is lower than that of ferrite [11,12]. Therefore, grain boundary ferrite (GBF) and side lath plate ferrite (FSP) will inevitably appear in weld metal. GBF and FSP tend to nucleate from proto-austenitic grain boundaries (PAGB) when the heat input is too high and the primary austenite grain is too large, the cooling rate is too slow, and the temperature stays too long in the high temperature region. Due to the different grain size, internal grain structure, and arrangement of AF, GBF, FSP, the mechanical properties of the welding area are also very different. Therefore, the mismatch between the mechanical properties of the three microstructures can easily produce stress concentration, which has a great impact on the stress field distribution, expansion path, and rate of the crack tip, thus reducing the fracture toughness [13,14,15,16].

Nowadays, there has been a lot of research on improving the fracture toughness of weld metal. Wang et al. found that the increase in Mn and Ni content would significantly increase the AF content and large-angle grain boundary density and improve the low-temperature toughness of the weld metal. However, the improper Mn and Ni content ratio will cross the martensite formation line, resulting in the formation of low-temperature phase transition products such as martensite and damage the low-temperature toughness [17]. X.N. Qi et al. found that with the increase in Ti content, the outer composition of inclusions in the weld was Al_2_O_3_→Ti_2_O_3_→TiC. Ti_2_O_3_ can promote the formation of manganese poor regions, which induce ferrite nucleation by increasing the chemical driving force, while Al_2_O_3_ and TiC do not work [18]. Z.Q. Wang et al. showed that the increase in Ni content can effectively reduce the ferrite transition temperature and reduce the proportion of GBF and FSP [19]. Kaiyue Liu et al. showed that the hardness difference between AF and GBF can be reduced after the post-welding heat treatment of the weld metal, thereby reducing the stress concentration between the phases. Post-welding heat treatment can also reduce dislocation density and turn dislocation entanglement into mobile dislocation, thus improving the low-temperature fracture toughness of the material [20].

In summary, current studies on improving the fracture toughness of weld metals mainly focus on reducing the heat input to inhibit the growth of GBF and FSP [10,21]. Alloying elements are regulated to reduce the austenite transition temperature, inhibit high-temperature transition products, and control the number and size of inclusions, thereby increasing the proportion of AF [17,19,22]. Post-welding heat treatment can reduce the stress difference between AF, GBF, and FSP and reduce the welding residual stress [23,24]. However, the influence mechanism of the three typical weld structures, i.e., AF, GBF, and FSP, on fracture toughness, especially on CTOD, is still unclear and lacks systematic research. In this paper, the microstructure information of AF, FSP, and GBF was deeply characterized and quantified via EBSD technology, and the fracture toughness of weld metal was evaluated via CTOD experiment, which elucidates the mechanism of the effect of the weld microstructure type on the fracture toughness of pipeline steel.

## 2. Experimental Section

### 2.1. Mechanical Properties and Welding Experiments of Base Metal

**Figure 1:**
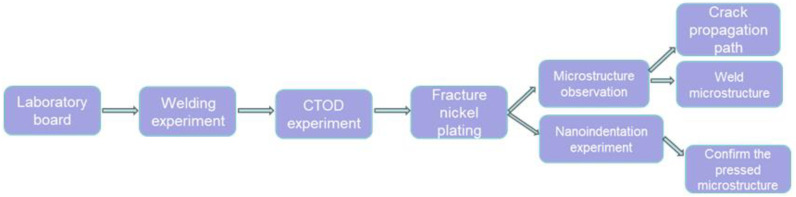
General process of experiment.

**Figure 2:**
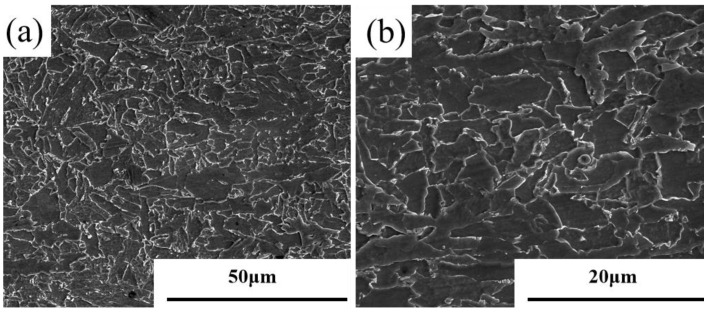
SEM characterization of X65 grade experimental steel: (**a**) 2000 times; (**b**) 5000 times.

The laboratory-made plate used in this experiment is X65 steel. Its chemical composition is shown in Table 1. The thickness of the steel plate is 30 mm. Figure 1 shows the SEM characterization of the base material. It could be observed from Figure 2a that the microstructure of the base material was mainly composed of granular bainite and ferrite, with a grain size of 5–16.5 μm. Figure 2b shows that the small M/A (martensite/austenite) components were dispersed at the ferritic grain boundaries. A rod-shaped tensile sample with a diameter of 5 mm and a length of 65 mm, oriented perpendicular to the rolling direction, was taken and subjected to room-temperature tensile testing according to the GB/T228.1-2021 [25] standard. The mechanical properties of the base material were shown in Table 2. The yield strength Rp0.2 was 493.5 Mpa, and the tensile strength Rm was 602.5 MPa.

Multi-wire submerged arc welding was used in laboratory welding experiments. H08MnMoTiB special submerged arc welding wire for pipeline steel is combined with flux SJ102G for welding. The composition of the welding wire is shown in Table 3. The welding speed was set at 1.10 m/min, and the heat input was varied by adjusting the current to a range of 1300–600 A and the voltage to 40–33 V. This resulted in the formation of five different microstructural ratios in the weld metal.

### 2.2. Crack Tip Opening Displacement Experiment (CTOD)

**Figure 3:**
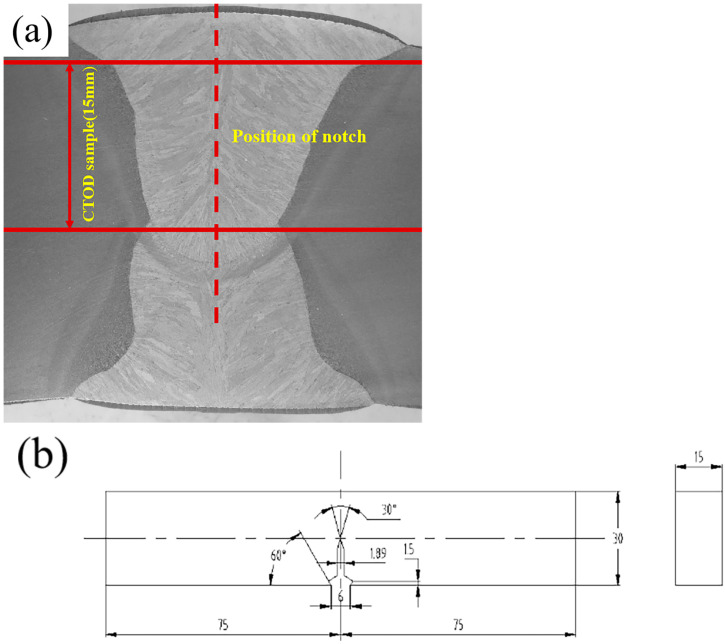
(**a**) Actual welded joints and sampling locations. (**b**) CTOD schematic diagram.

The CTOD experiment sample was a three-point bending standard sample. A 150 mm × 30 mm × 15 mm block sample was taken from the center of the weld seam, and a mechanical notch was made at the center of the weld seam as shown in Figure 3. The sampling position is shown in Figure 3a. A mechanical notch was made in the center of the weld (as shown by the dashed line in Figure 3a), as depicted in Figure 3b. The fatigue crack was prefabricated by a fatigue experimenting machine at room temperature, and the initial crack length was 13.2–14.26 mm. According to GB/T-21143-2014 standard, the crack tip opening displacement experiment was carried out with GNT200 universal experimenting machine (NCS, Beijing, China) under the condition of −10 °C, and three sets of parallel experiments were carried out for each weld. The CTOD value was calculated according to GB/T-21143-2014 [26] standard, and the formula is as follows:

(1) $$\delta ={\left[(\frac{S}{W}\frac{F}{{\left(BB_{N}W\right)}^{0.5}}\times g_{1}\left(\frac{a}{W}\right)\right]}^{2}\left[\frac{\left(1-v^{2}\right)}{2R_{p0.2}E}\right]+\frac{\left(R-a-Z\right)V_{p}}{R}$$

where F (N) is the critical load, E (MPa) is Young’s modulus, ν is Poisson’s ratio, and R_p0.2_ (MPa) is the yield strength of the material. W, B, a, and Z are specimen dimensions (mm). For the sample without side groove, BN = B, g_1_ (a/w) is the relevant parameter S, and R is the working distance (mm).

### 2.3. Observation of Microstructure and Crack Growth Path

Due to the many uncertain factors in the welding process, in order to ensure accurate matching between the CTOD experiment results and the weld microstructure, this study used the samples after the CTOD experiment to observe the microstructure of the weld metal. The base metal part of the CTOD experiment sample was cut off, and then it was split in half along the direction perpendicular to the section to observe the influence of different types of microstructures on the crack propagation path. In order to ensure that the complete morphology of the crack propagation path was not damaged during the cutting process, the fracture area was protected by nickel plating before it was cut. The cut sample was embedded using conductive inlay material at 150 °C for 15 min using a hot inlay machine. The embedded sample was polished in four stages using 150–1000 grit sandpaper. Then, a polishing machine was used to polish it until it was smooth and free of scratches. Finally, the sample surface was soaked in a 4% nitric acid ethanol solution for 15 s to corrode it. The cross-section of corroded weld metal was observed using Olympus GX51 optical microscope (OM) and FEIQuanta650FEG thermal field emission scanning electron microscope (Fremont, CA, USA). The surface of the sample was polished using the vibration polishing method, and the crystallographic information in the weld metal was obtained by a OxfordF plus backscatter diffractometer via field emission scanning electron microscopy (NCS, Beijing, China). The EBSD data were processed and analyzed by HKL-Channel 5 2019 v5.12 software so as to obtain the required inverse pole figure (IPF), grain boundary map, and MBS diagram. The ImageJ V1.8.0.112 software was used to statistically analyze the proportion of different microstructures, grain size, and aspect ratio in the weld metal.

### 2.4. Nanoindentation Experiment

We used a hot setting machine to set the sample height to 30 mm. After polishing the embedded sample, the 5100 AFM multi-functional scanning probe (Agilent, Santa Clara, CA, USA) was used to conduct nanoindentation experiment at 2 mm below the crack growth path to measure the hardness of the single-phase microstructure. We pressed down a 10 × 10 matrix in an S-shaped upward arrangement, with a maximum load (Pmax) of 200–300 mN, a loading time of 15 s, and a load-holding time of 10 s. After the experiment, the sample was soaked in 4% nitrate ethanol for 5 s for mild etching and observed under FEIQuanta650 thermal field emission scanning electron microscope to confirm the loading position and eliminate error data.

## 3. Experimental Results

### 3.1. CTOD Experiment Results

The results with large deviations in the three parallel experiments were deleted, the results of the two groups of experiments were retained, and the average value was obtained. The final results were numbered from low to high according to the average value, as shown in Table 4. It can be observed that the critical CTOD value δ_c_ of the weld fluctuates between 0.2 and 0.6 mm, of which the highest critical CTOD value of the 5# weld is 0.582 mm on average, and the lowest critical CTOD value of the 1# weld is 0.237 mm, which is only 40% of the 5# weld.

### 3.2. Microstructure of Weld Metal

**Figure 4:**
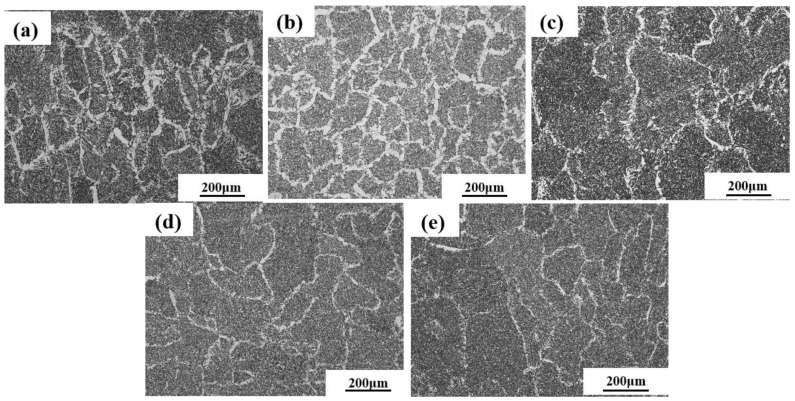
Microstructure of 1#–5# weld metal at 100 times (**a**–**e**), respectively.

**Figure 5:**
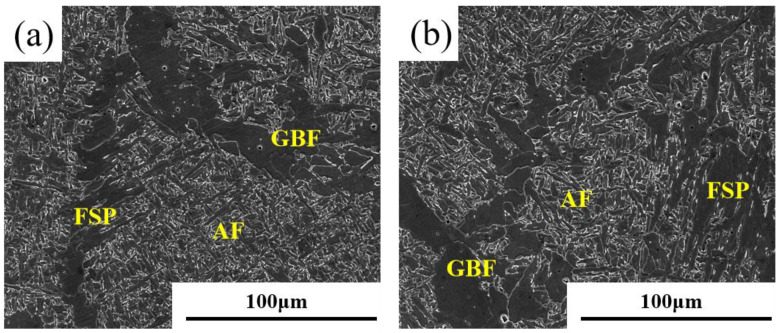
SEM characterization of AF, FSP, and GBF in weld metal. (**a**) 5# weld metal; (**b**) 1# weld metal.

Figure 4 shows the metallographic micrographs of five kinds of weld structures; their corresponding critical CTOD values are 1# (0.222 mm), 2# (0.264 mm), 3# (0.390 mm), 4# (0.474 mm), and 5# (0.599 mm), respectively. It can be seen that the microstructure of the weld had mainly been composed of non-directionally arranged AF, thick large layers of FSP inserted into the protoaustenite crystals, and GBF growing along the protoaustenitic grain boundary. The statistical results of its proportion, obtained using ImageJ software, are shown in Figure 5. AF, as the microstructure with the greatest impact on the mechanical properties of weld metal, accounts for approximately 65–94.61%. GBF and FSP were harmful structures in weld metal, accounting for 0.59–26.1% and 3–27.1%, respectively.

Figure 5 shows the SEM characterization of the weld metal. A more detailed characterization of the weld microstructure via a scanning electron microscope shows that AF is closely arranged in a braided form, with a relatively small grain size and a width of only 0.7–2.7 μm. GBF is an axisymmetric massive structure, nucleated from the grain boundaries of the original austenite in a network arrangement, with large grain size. FSP nucleates from the grain boundary of the original austenite, the lamellae penetrates into the inner grain of the original austenite to destroy the interlock structure of AF, and the aspect ratio can generally reach 20 or more.

**Figure 6:**
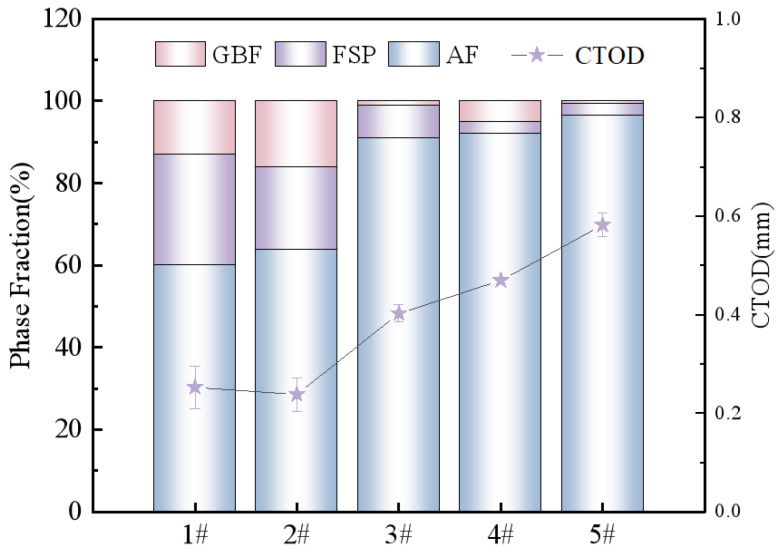
Comparison of microstructure ratio and CTOD value in 1#–5# welds.

As shown in Figure 6, the CTOD experimental results were compared with the proportion of the microstructure in the weld metal to analyze the influence of different microstructures on fracture toughness. It can be observed that the proportion of AF plays a decisive role in the fracture toughness of the weld. With the increase in AF, the CTOD value of the weld metal is higher. Further grouping and comparing the microstructures of welds 1# and 2# with an AF ratio of approximately 65% and welds 3# and 4# with an AF ratio of approximately 90%, it was found that when the AF ratio was basically the same, the higher the FSP ratio, the lower the CTOD value. This indicates that FSP has a higher impact on fracture toughness than GBF.

### 3.3. Results of Nanoindentation Experiment

**Figure 7:**
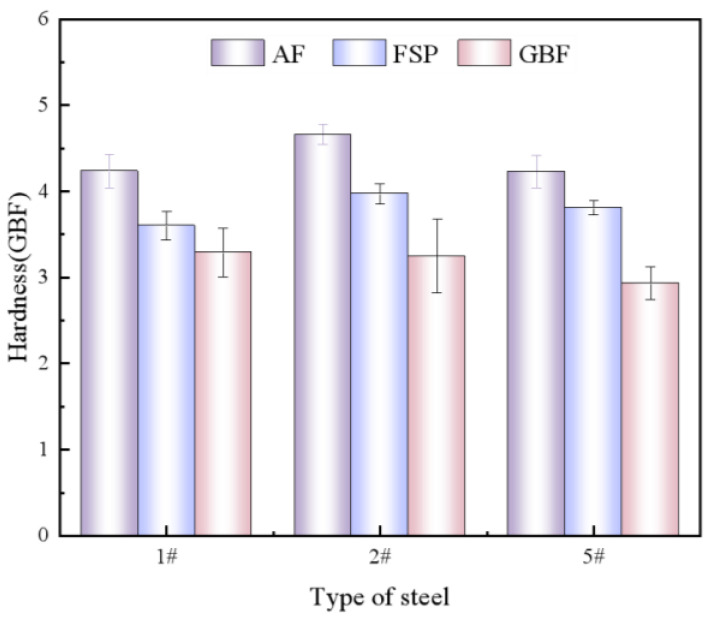
Nanoindentation experiment results of AF, FSP, and GBF in 1#, 2#, and 3# weld metals.

To compare the hardness of single-phase structures in AF, GBF, and FSP, nanoindentation tests were conducted on the microhardness of single-phase structures in the 5# weld with high AF content, the 2# weld with high GBF content, and the 1# weld with high FSP content. The experimental results are shown in Figure 7, and it can be observed that there is a significant difference in hardness between the three tissues. AF is the microstructure with the highest hardness in the weld seam, ranging from 4.23 to 4.66 GPa. GBF, as the soft phase in the weld seam, has a hardness value of 2.92–3.29 GPa, which is about 30% lower than the hardness of AF. The hardness of FSP ranges from 3.6 to 3.97 GPa between AF and GBF.

## 4. Discussion

### 4.1. Influence of Weld Microstructure on Fracture Toughness

**Figure 8:**
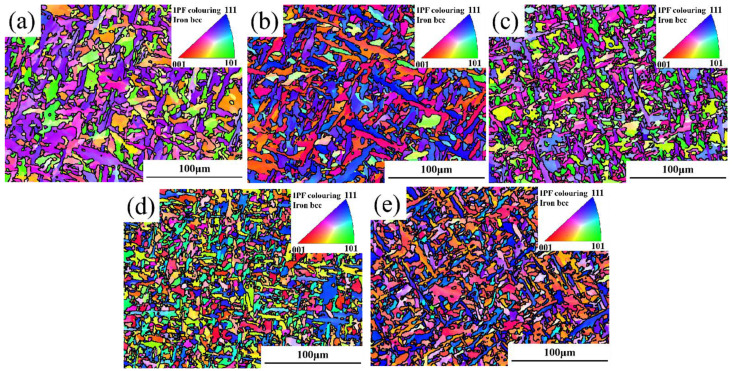

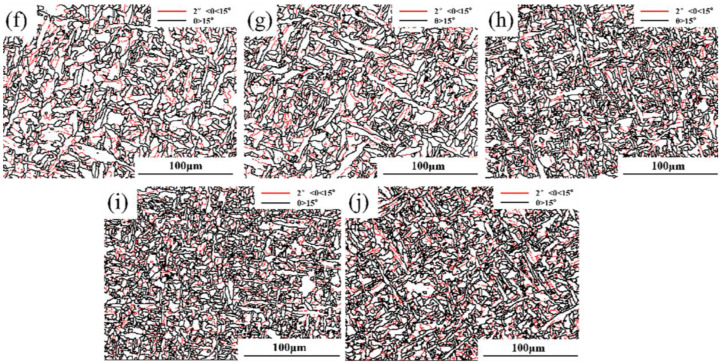
EBSD characterization of AF in different welds: (**a**–**e**) are IPF diagrams of AF in 1#–5# welds; (**f**–**j**) are grain boundary diagrams of AF in 1#–5# welds.

Because EBSD technology is extremely sensitive to grain internal stress and microstructural defects, this study aims to further investigate the influence of weld metal microstructure on fracture toughness. EBSD technology is used to finely characterize AF, GBF, FSP, and crack propagation paths in weld metal. Figure 8a–e show the inverse pole diagram (IPF) of AF in five kinds of welds, and the different colors of the scale of the triangle on the top right of the picture represent three different orientations. The IPF diagram shows that the crystal orientation of adjacent AF grains is different, the orientation difference between adjacent grains is large, and the overall orientation distribution is uniform. Figure 8f–j are the grain boundary diagrams of AF. If the orientation difference between adjacent grain boundaries is greater than 2° and less than 15°, it is set as a low-angle grain boundary (LABG) in red; if the orientation difference between adjacent grain boundaries is greater than 15°, it is set as a high-angle grain boundary (HAGB) in black. According to statistics, the grain boundary energy of AF is relatively high, which is basically a HAGB greater than 45°. Therefore, when cracks pass through AF, more crack propagation energy is consumed, which can significantly reduce the crack propagation rate [27].

**Figure 9:**
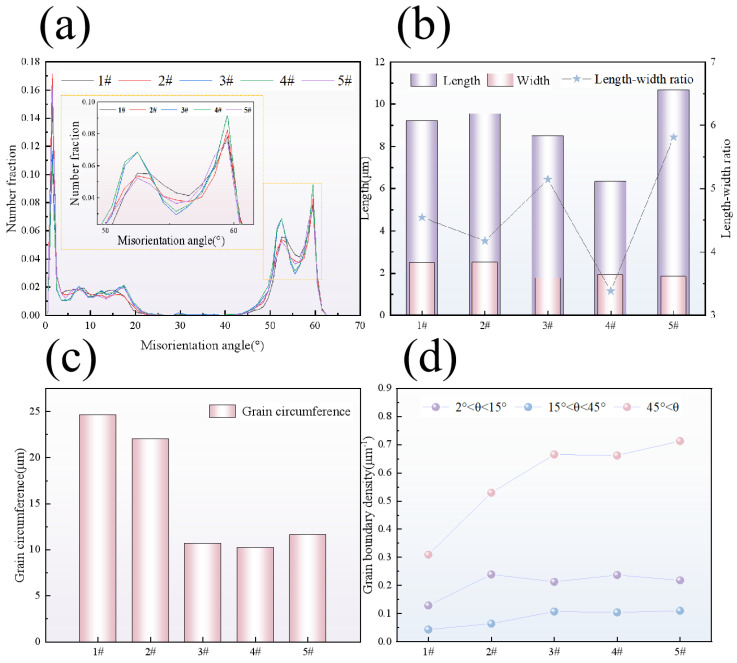
AF crystal information analysis in 1#–5# weld metal: (**a**) distribution diagram of orientation difference angle; (**b**) aspect ratio chart; (**c**) grain circumference map; (**d**) grain boundary density map.

Previous studies have shown that the larger the proportion of the HAGB, the stronger the anti-crack propagation ability of the material [28,29]. However, it can be observed from the AF orientation difference angle distribution diagram of the five welds in Figure 9a that the AF grain of the 5# welds with the highest CTOD value (0.599 mm) has the lowest HAGB ratio. This cannot reasonably explain the results of this CTOD experiment. Therefore, in this study, 200 AF grains were selected from each weld for the aspect ratio; grain circumference statistics and the grain boundary density per unit area were calculated to study the influence of AF on the fracture toughness of the weld. Figure 9b shows the statistical diagram of the aspect ratio of AF grains in five kinds of welds. It can be seen that the aspect ratio of AF is not significantly correlated with the change in CTOD value. Figure 9c shows the statistical graph of effective grain perimeter. It can be seen that the effective grain perimeter decreases significantly as the width of the AF grain slat becomes narrower. Combined with the grain boundary diagram of the angle size, it can be seen that AF grains are obviously refined and CTOD values are obviously increased with the decrease in grain circumference. Since the number of large-angle grain boundaries between different welds cannot be compared in the orientation difference distribution diagram, the grain boundary densities of 2~15°, 15~45°, and larger than 45° in the AF of the five groups of welds were calculated. It can also be seen from the grain boundary density diagram in Figure 9d that with the decrease in the grain perimeter, the grain boundary densities of 2~15° and 15~45° per unit area have no significant change, but the grain boundary density of those greater than 45° increases by about 49.6%.

With the decrease in grain circumference and the increase in grain boundary density greater than 45°, the large-angle grain boundary per unit area increases obviously, and the grain size of the AF also shows obvious refinement. Grain refinement can significantly increase the density of large-angle grain boundaries, thereby enhancing the ability to hinder dislocation slip and improving the strength and toughness of weld seams [30]. When the grain size of the AF material becomes finer, it is difficult for microcracks to penetrate straight through the fine and tightly interwoven AF strip bundle, which effectively provides more obstacles for crack propagation and thus significantly delays the propagation rate of main cracks. In summary, the refinement of the grain size of AF can greatly improve the fracture toughness of the weld so that the weld has a stronger ability to resist crack growth in the face of external forces.

**Figure 10:**
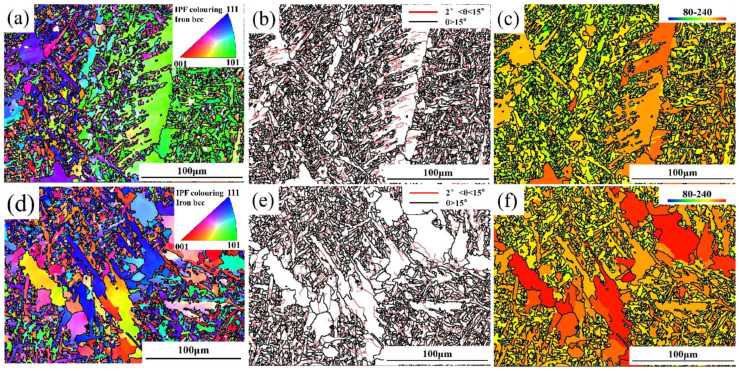
EBSD characterization of 1# and 5# weld metals: (**a**–**c**) IPF diagram, grain boundary diagram, and MBS diagram of 5# weld metals; (**d**–**f**) IPF diagram, grain boundary diagram, and MBS diagram of 1# weld metal.

It can be seen from Figure 6 that when the ratio of AF in the weld plays a decisive role in the fracture toughness of the weld but the ratio of AF is basically the same, the higher the ratio of FSP, the lower the CTOD value. To investigate the mechanism of FSP and GBF on the fracture toughness of weld metal, the 1# and 5# weld metals with a large difference in the proportion of FSP and GBF were characterized via EBSD, and the results are shown in Figure 10. It can be observed from the IPF diagram in Figure 10a,d that the grain size of FSP in the 5# weld is obviously smaller than that of the 1# weld, that the lamellar width is narrow, and that FSP does not penetrate too deeply into the original austenite grain, destroying the interlock structure of AF. The layer width of FSP in the 1# weld is 2.7 μm, and the length is 31.2 μm, which significantly damages the interlocking structure of AF and provides a path for the crack to break through the obstacle of AF. Comparing the grain interior of FSP and GBF in Figure 10b,e, it is found that the grain interior of GBF is relatively clean, while there is an obvious LAGB in FSP, which mainly reflects the substructure and dislocation density inside the grain. The dislocation density in FSP is significantly higher than that in GBF. Due to dislocation movement and entanglement during deformation, it is easy for there to be accumulations at the grain boundary, resulting in stress concentration, which provides the nucleation site for crack initiation and reduces the energy required for crack nucleation.

Figure 10c,f shows the average band slope plot (MBS) of grains, where the MBS value is defined as the slope of the intensity variation between the background of the Kikuchi pattern and the band. MBS describes the quality of the Kikuchi band, and the lower the MBS value, the poorer the quality of the Kikuchi band, indicating that there are more defects inside the grains [31,32]. The MBS value in the HL-Channel5 2019 v5.12 software ranges from 0 to 300. In this study, based on the characteristics of the weld microstructure, the range of MBS value is set from 80 to 240. The MBS value of the microstructure is reflected by the color, and the color band in the upper left corner is used as the scale. It can be observed from Figure 10c,f that the MBS value of FSP is significantly lower than GBF, indicating that the dislocation density and internal stress inside the grain are higher than GBF, which corresponds well to LAGB in FSP in Figure 10b,e. The different formation mechanisms of FSP and GBF will also affect the mechanical properties. Although both GBF and FSP are high-temperature phase transition products generated along the grain boundary of the original austenite, the formation mechanisms of the two are fundamentally different. Different from GBF’s single diffuse transition, FSP also has some lattice shear, which is the same as the formation mechanism of ferrite in bainite. The diffusive transition often presents uniform or gradual change characteristics, so the stress field generated by it is relatively simple. Lattice shear can lead to local changes in the crystal structure, such as distortion or deformation of the crystal lattice, which will produce a stress field within the grain, resulting in greater internal stress within the grain. In summary, more LAGB and internal stress in FSP grains will lead to increased lattice distortion energy and more crack nucleation sites in local stress concentration, thus inducing crack initiation. Therefore, FSP has greater damage to WM’s fracture toughness than GBF [24,33,34].

From the microhardness values of AF, FSP, and GBF in 1#, 2#, and 5# obtained via nanoindentation experiment in Figure 7, it can be seen that there is an obvious hardness difference between the three microstructures. Both FSP and GBF are soft phases with low yield points, while AF is a hard phase with high toughness and strength, which seriously damages the continuity of the mechanical properties of the weld. The mismatch of mechanical properties between the three kinds of structures will lead to the uneven distribution of strain and stress in the deformation process of WM, so the plastic strain localization of GBF and FSP is prone to occur in the deformation process, forming crack sources [20,35,36].

### 4.2. Influence of Weld Microstructure on Crack Propagation Path

**Figure 11:**
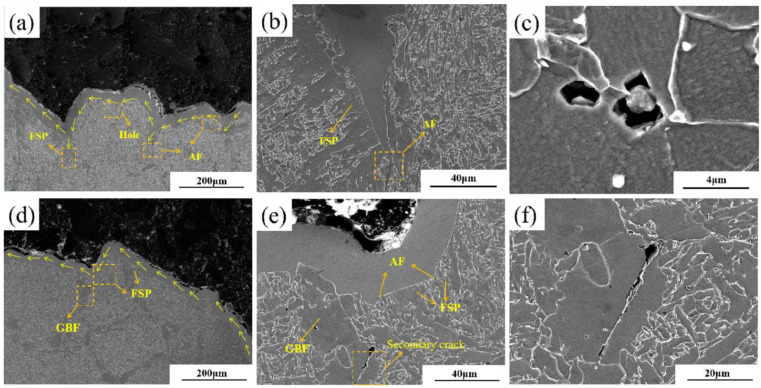
SEM characterization of the crack trend in the brittle fracture zone of 1# and 5# weld metals: (**a**) the crack trend diagram of 5# weld metals; (**b**,**c**) a partial enlargement of (**a**); (**d**) diagram of the crack pattern of 1# weld metal; (**e**,**f**) a partial enlargement of (**d**).

In order to clearly observe the influence of the microstructure of the weld on the crack growth path, the pattern was split in half along the direction perpendicular to the section, and the crack growth path was characterized. Figure 11a,d shows the crack growth paths in the brittle region of 5# and 1#, respectively. It can be seen that the crack propagation path of weld 1# is relatively straight, with fewer turning points; while the crack propagation path of weld 5# is more tortuous, with a longer total crack length. As shown in Figure 11b,e, it can be observed by magnifying the turning point that cracks expand in a straight line when passing FSP and GBF, which consumes less crack propagation energy, accelerates the crack propagation rate, and seriously damages the fracture toughness of weld metal. When the crack passes through AF, the crack has obvious deflection, and most of the deflection angle is greater than 90°. From Figure 11c, it can be observed that there are many micropores distributed in the area near the main crack. The formation of these micropores is due to the shedding phenomenon caused by the stress difference between the inclusion and the matrix. These micropores will become the nucleation sites of cracks, and AF has good plastic deformation ability, which inhibits the further expansion of micropores and reduces the risk of ductile fracture caused by micropore polymerization. This mechanism effectively enhances the crack stopping ability of the weld metal and then significantly improves the fracture toughness of the weld metal. As shown in Figure 11f, there are more secondary cracks in the matrix below the main crack of the 1# weld, and the secondary cracks mostly appear in GBF and FSP, which are linear transgranular fractures. The cracks stop at the grain boundary of AF, and the length is related to the size of the grain where they are located. When the secondary crack occurs in the AF, the total length is shorter and the deflection at the grain boundary is obvious.

**Figure 12:**
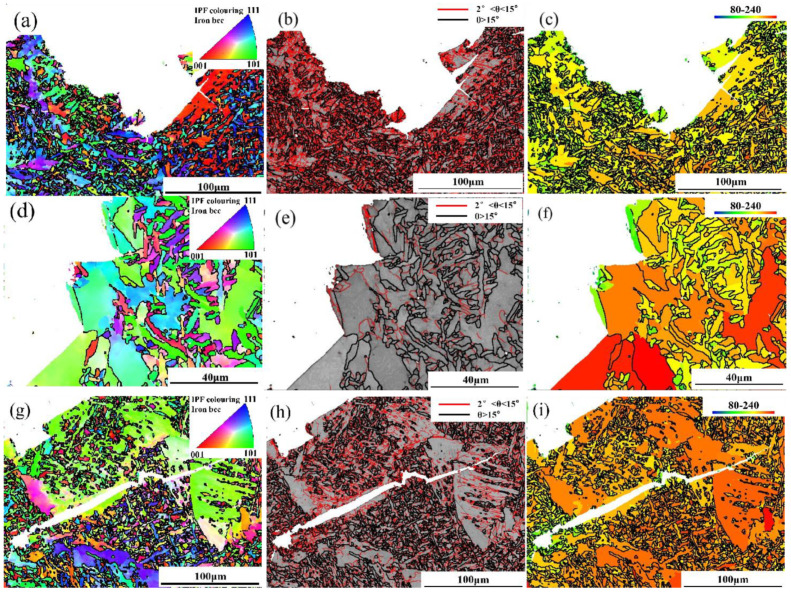
EBSD characterization of crack trend in the brittle fracture zone of 1# and 5#WM: (**a**–**c**) IPF diagram of 5# weld metal, grain boundary diagram of size and angle, and MBS diagram; (**d**–**f**) IPF diagram of 1# weld metal, the grain boundary diagram of size and angle, and the MBS diagram; (**g**–**i**) IPF diagram, the grain boundary diagram, and the MBS diagram of the secondary cracks in the 1# weld metal matrix.

In order to deeply explore how AF, GBF, and FSP in weld metal affect the crack propagation path, respectively, as well as the root cause of secondary crack initiation, EBSD characterization was performed on the microstructure at the crack site, as shown in Figure 12. It can be observed from Figure 12a that due to the large grain boundary energy of a crack passing through AF, more crack growth energy will be consumed to break the barrier of HAGB (≥45°). Moreover, the HAGB density is high, which will effectively promote the frequent deflection of cracks until the cracks stop expanding, thus improving the fracture toughness of the weld metal. As a soft phase in weld metal, GBF will accumulate more plastic deformation due to the uneven distribution of stress and strain during deformation. At the same time, the hardness difference between GBF and the adjacent AF matrix is large, resulting in stress concentration. Therefore, it can be seen from Figure 12d–f that when the crack passes through GBF, the crack will preferentially expand along the grain boundary from GBF. It can be seen from the secondary cracks on the matrix that crack nucleation in FSP is mainly due to the accumulation of dislocations, and crack nucleation is the critical point of the entire fracture process. After nucleation at the FSP grain boundary, the crack rapidly grows unstable [37]. Figure 12g–i shows the typical transgranular fracture in FSP and GBF, indicating that the energy consumption of crack growth in FSP and GBF is small, and the ability to prevent cracks is poor. There are many substructures and dislocations in FSP which not only provide nucleation sites for cracks but also promote the rapid propagation of cracks. When the secondary crack passes through AF, the crack is deflected and terminated, which effectively prevents the expansion of the secondary crack.

## 5. Conclusions

1. The weld microstructure of the steel suspension chain riser is mainly composed of AF, FSP, and GBF. The CTOD experimental results are positively correlated with the proportion of AF, indicating that an increase in the proportion of AF will correspondingly enhance fracture toughness. When the proportion of AF remains relatively stable, the ability of weld metal to resist crack propagation will weaken with the increase in FSP proportion.
2. AF, as the main microstructure in weld metal, plays a decisive role in fracture toughness. AF grain boundaries are large-angle grain boundaries with an orientation difference greater than 45°. They have high grain boundary energy and can effectively hinder crack propagation. As the proportion of AF in the weld metal increases and the effective grain size is refined, the grain boundary density of the HAGB can be significantly improved, thereby enhancing the fracture toughness of the weld metal.
3. The large difference in hardness between GBF and AF results in unstable mechanical weld metal properties. As a soft phase, the GBF phase interface is prone to becoming a nucleation site for cracks, and the grain boundary energy is low. When cracks pass through GBF, it exhibits less resistance to crack propagation due to intergranular fracture.
4. Compared with GBF, FSP has a larger grain size and destroys the interlocking structure of AF in a layered manner. Additionally, there are more substructures within the grain, leading to concentration of internal stress and reducing the fracture toughness of the weld.

## Figures and Tables

**Table 1:** Chemical composition and content of X65 grade experimental steel.

Element	C	Si	Mn	P	S	Cr	Ni	Mo	Cu	Nb	Ti	V	Al
content Wt%	0.049	0.18	1.18	0.003	0.0016	0.17	0.2	0.09	0.1	0.024	0.014	0.023	0.03

**Table 2:** Mechanical properties of base metal.

Pipeline	R_t0.2_/Mpa	R_m_/Mpa	R_t0.2_/R_m_	A/%
X65	493.5	602.5	0.82	26

**Table 3:** Chemical composition of welding wire.

H08MnMoTiB	C	Si	Mn	P	S	Mo	Ti	B
Content Wt%	0.11	0.16	1.86	0.008	0.005	0.32	0.06	0.004

**Table 4:** CTOD results of 1#–5# weld metal.

Number	Critical CTOD Experiment Results		Mean Value
1#	0.222	0.252	0.273
2#	0.214	0.26	0.238
3#	0.390	0.415	0.403
4#	0.465	0.474	0.469
5#	0.565	0.599	0.582

## Data Availability

The original contributions presented in the study are included in the article, further inquiries can be directed to the corresponding author.
